# Association between a frequent variant of the *FTO* gene and anthropometric phenotypes in Brazilian children

**DOI:** 10.1186/1471-2350-14-34

**Published:** 2013-03-13

**Authors:** Carmela Farias da Silva, Marília Remuzzi Zandoná, Márcia Regina Vitolo, Paula Dal Bó Campagnolo, Liane Nanci Rotta, Silvana Almeida, Vanessa Suñé Mattevi

**Affiliations:** 1 Programa de Pós-Graduação em Patologia, Universidade Federal de Ciências da Saúde de Porto Alegre, Porto Alegre, RS, Brazil; 2 Programa de Pós-Graduação em Ciências da Saúde, Universidade Federal de Ciências da Saúde de Porto Alegre, Porto Alegre, RS, Brazil; 3 Universidade Federal de Ciências da Saúde de Porto Alegre, Rua Sarmento Leite, 245, sala 309, Porto Alegre, RS, CEP 90050-170, Brazil

**Keywords:** Childhood obesity, *FTO* gene and genetic susceptibility

## Abstract

**Background:**

Our goal was to analyze the association of the fat mass and obesity- associated *(FTO)* gene rs9939609 variant (T/A) with the anthropometric and dietary intake phenotypes related to obesity in Brazilian children.

**Methods:**

We analyzed the association of this single nucleotide polymorphism (SNP) with phenotypes related to the accumulation of body mass in a cohort of 348 children followed from the time of birth until 8 years old and then replicated the main findings in an independent schoolchildren sample (n = 615).

**Results:**

At the age of 4, we observed a significant association between the A/A genotype and a higher mean BMI Z-score (P = 0.036). At the age of 8, the A/A individuals still presented with a higher BMI Z-score (P = 0.011) and with marginal differences in the volume of subcutaneous fat (P = 0.048). We replicated these findings in the schoolchildren sample, which showed that those with at least one copy of the A allele presented with a higher BMI Z-score (P = 0.029) and volume of subcutaneous fat (P = 0.016).

**Conclusion:**

Our results indicate that this *FTO* variant is associated with increased body mass and subcutaneous fat in Brazilian children beginning at the age of 4.

## Background

Childhood obesity is an increasing public health problem all over the world, including many low- and middle-income countries. This complex disease is a major cause of morbidity and mortality; a higher risk of obesity is associated with premature death and disability in adulthood. According to the World Health Organization (WHO), 1.6 billion adults (aged 15 years and older) were overweight and 400 million were obese in 2005. By 2015, these figures are predicted to rise to 2.3 billion overweight adults and over 700 million obese adults [1].

Characteristics of the Western lifestyle such as excessive energy intake and low levels of physical activity largely influence the increasing prevalence of obesity worldwide. In addition to environmental factors, genetic factors also clearly contribute to obesity-related phenotypes, with heritability estimates ranging from over 50% to 60% for body mass index (BMI) [2].

A recent Genome Wide Association study (GWAs) for type 2 diabetes mellitus (T2DM) susceptibility genes identified common variants in the *FTO* gene (*fat mass and obesity associated*) that predispose adults to diabetes through an effect on BMI [3]. Several single nucleotide polymorphisms (SNPs) have been described in this gene, but a T-to-A change in intron 1 (rs9939609) is the most widely investigated and is consistently associated with obesity-related phenotypes, mainly body mass index (BMI). Approximately 63% of all individuals of European origin carry at least one A - risk allele of this *FTO* variant, 16% being A/A homozygotes [4]. The risk of being obese correlated to each A allele ranges from 1.35 to 1.76 [5].

Of the many tissues in which the *FTO* gene is highly expressed, the hypothalamic nuclei are of particular interest because the nuclei control energy balance and are influenced by nutritional signals [6,7]. However, the exact role of the *FTO* gene product in the regulation of human energy metabolism is still unclear. While there is some evidence indicating that the *FTO* gene product functions during early infancy [3], many questions persist regarding the timing of gene function and what systems are affected.

Multiple studies have associated *FTO* polymorphisms with obesity in different European populations, thereby making this gene one of the most important loci implicated in polygenic obesity thus far [8]. However, the contribution of the *FTO* common variants to obesity is controversial in African American, Asian (Chinese Han and native Oceanic) and Hispanic populations [9-13]. To our knowledge, there are no studies in Brazil addressing the effect of *FTO* variation on BMI in children.

In the present study, we analyzed the association between the *FTO* rs9939609 gene variant and predisposition to weight gain by profiling excessive accumulation of body mass and diet composition in a cohort of children followed from the time of birth until the age of 8 years old. The major findings were then replicated in another independent sample of children.

## Methods

### Cohort sample

In a previous study conducted by our group, 500 mother-child pairs were recruited at birth between October 2001 and July 2002 at Centenario Hospital, the only publicly funded hospital of São Leopoldo, RS, Brazil, and randomized into intervention and control groups. The intervention group followed a program of dietary guidelines: “Ten steps to healthy eating for children under two years” [14]. The intervention consisted of dietary advice about breast-feeding and adequately introducing complementary foods. The intervention group was monitored with home visitations within 10 days of the child’s birth, then on a monthly basis up to 6 months and subsequently at 8, 10 and 12 months. Trained fieldworkers provided dietary counseling to the mothers based on the Ten Steps. No subsequent intervention was performed. The prerequisites for the study were a birth weight heavier than 2500 grams and a gestational age of more than 37 weeks. Mother-child pairs were excluded if they possessed any of the following characteristics: HIV positive mother, congenital malformation, infant referred to the Intensive Care Unit and multiple deliveries. The parents reported their ethnicity according to their skin color as white or non-white, which follows the standards of the official population censuses in the country.

The children who completed the first phase of the study (n = 397) were reevaluated at the age of 3 to 4 years old at home by trained interviewers to collect food and anthropometric data [15]. Blood samples for biochemical and genetic analyses were also collected at this time. Informed consent was obtained from the parents, and the protocol was approved by the Ethics Committee of the Federal University of Health Sciences of Porto Alegre, n° 736/08.

After reaching the age of 7 to 8 years, the same children were again assessed for dietary, metabolic and anthropometric data (n = 308), and blood samples were collected for biochemical measurements.

### Dietary patterns

At 12 to 16 months of age, undergraduate nutrition students recorded the child’s food intake for a 24-hour period prior to the home visit. When the children turned 3 to 4 years old, the dietary patterns were recorded from the food intake diaries for two randomly selected and nonconsecutive 24-hour periods, the first performed at home and the second during the ambulatory evaluation. To quantify the food portion size, pictures were used to illustrate units of measurement, such as teaspoons, tablespoons, cups and food portion.

At 7 to 8 years old, the children were revisited in their homes to assess their diet. Two food intake diaries were recorded over a 24-hour period: the first was documented at home and the second was performed along with anthropometric measurements at school.

The nutritional values for the diets were calculated using the NutWin (Nutritional Support Program from the Federal University of São Paulo), food chemical composition tables [16,17], and information obtained from food industries for products not included in the tables. The mean food intake from the two dietary diaries at 3 to 4 years and at 8 years old was used for analysis.

### Anthropometry

The anthropometric data were collected during three phases. During the first phase, between 12 and 16 months of age, the weights of the children were measured using a portable digital scale (Techline®; São Paulo, Brazil), and their heights were measured using a portable stadiometer (Seca®; Hamburg, Germany) with the children dressed in light clothing and no shoes. At 3 to 4 years old, the height, weight, tricipital and subscapular skinfold thickness and waist circumference were measured during the second phase of data collection. The sum of the two individual skinfolds was computed. The body mass index (BMI) was calculated, [weight(kg)/height(m)^2^], and the values were transformed into a Z-score according to the WHO Growth Standards specific for sex and age. When the children reached 8 years, new anthropometric evaluations were performed. The methodology used was the same as that described above.

### Replication sample

To replicate the previous findings, a total sample of 615 school children and adolescents (4 to 18 years old) was used in this work. This sample is part of a cross-sectional study performed in Sapucaia do Sul, RS, Brazil, which was approved by the Ethics Committee of the Federal University of Health Sciences of Porto Alegre, n° 792/11.

After the parents provided written informed consent, blood samples (5 mL) were collected, and data regarding age, gender and phenotypic characteristics were obtained through a questionnaire that was recorded on the same day on which the blood samples were collected. The anthropometric data were collected as previously described.

### Molecular analyses

DNA was extracted from whole blood using the previously described method of precipitation with a high salt concentration [18].

The *FTO* variant rs9939609 was genotyped through real-time polymerase chain reaction with the use of an allele discrimination assay (TaqMan SNP genotyping assay) from Applied Biosystems (Foster City, CA).

### Statistical analysis

The allele and genotype frequencies of rs9939609 were compared among ethnic groups, and the chi-square test was used to test their agreement with Hardy-Weinberg equilibrium in each group separately.

The mean anthropometric and dietary variables were compared between genotypes using analysis of variance (ANOVA) when normally distributed. The homogeneity of variances among the genotypic groups had previously been tested for each variable by the Levene’s statistic, and non-parametric tests were used when appropriated. When ANOVA provided significant results, the Tukey *post-hoc* test for multiple comparisons was used.

The variables with non-normal distribution, such as the sum of skinfolds, were logarithmically transformed for analyses or compared by Kruskal-Wallis test when necessary.

The mean percent BMI variation between 12 to 16 months and 3 to 4 years of age was compared among genotypes and adjusted for the covariates of sex, age and intervention or control group, using General Linear Models (GLM). The BMI variation between 4 and 8 years of age was also compared among genotypes using a similar method.

Multilevel mixed models were also used to analyze genotype effects on the patterns of individual change in anthropometric and dietary variables over time, as described in [19,20].

All statistical analyses were performed with SPSS version 20.0 for Windows software, and the differences were considered significant when P < 0.05.

## Results

The genotypic frequencies (Table 1) agreed with those expected under Hardy-Weinberg equilibrium (P = 0.099 for whites and P = 0.879 for non-whites). The allelic frequencies (A = 40%) were similar to frequencies in other studies with European descendants [3,21,22].

**Table 1:** **Genotype and allele frequencies of *FTO *gene SNP rs9939609**

**Genotype**	**Frequencies, n (%)**	**Ethnicity**		***P *Value**
		**White, n (%)**	**Non-white, n (%)**	
**T/T**	130 (37.4)	59 (42.4)	63 (34.6)	0.273^1^
**T/A**	161 (46.3)	56 (40.3)	89 (48.9)	
**A/A**	57 (16.4)	24 (17.3)	30 (16.5)	
**A Alelle Frequency**	0.40	0.37	0.41	0.410^1^

^1^ Chi-Square test.

The total sample was stratified between whites and non-whites. No statistically significant differences were observed between these groups for allelic and genotypic frequencies (Table 1; P = 0.410 and P = 0.273, respectively). No differences were observed regarding either anthropometric or dietary measurements among ethnicities, also. So, the individuals from both ethnicities were grouped in further analyses.

The mean anthropometric and dietary variables were compared among the different genotypes at the ages of 1, 4 and 8 years old (Table 2). There were no significant differences in phenotypes between genotypes at 1 year of age. At the age of 4 years old, we observed a statistically significant difference in the BMI Z-score among the three genotypic groups (P = 0.036). After post-hoc analysis, we found that the A/A individuals possessed a higher mean BMI Z-score than the T/A individuals (P = 0.028). There was no evidence of an association between the *FTO* genotypes and the sum of skinfolds, the waist circumference, the total energy intake and the percent BMI variation between the ages of 1 and 4 years old.

**Table 2:** **Mean anthropometric parameters and dietary patterns according to *FTO *genotypes in the São Leopoldo cohort**

**Phenotypes**	**T/T**		**T/A**		**A/A**		***P***
	**Mean ± SD**	**n**	**Mean ± SD**	**n**	**Mean ± SD**	**n**	
**1 year**							
**BMI Z-score**	0.57 ± 1.05	129	0.55 ± 1.02	157	0.70 ± 1.22	56	0.615^1^
**Total energy intake (kcal/day)**	946 ± 369	122	924 ± 422	144	891 ± 405	54	0.734^2^
**Energy intake from lipids (kcal/day)**	261 ± 120	122	255 ± 138	144	238 ± 142	54	0.567^1^
**Energy intake from carbohydrates (kcal/day)**	528 ± 221	122	517 ± 240	144	507 ± 216	54	0.830^1^
**4 years**							
**BMI Z-score**	0.26 ± 1.19^6,7^	125	0.13 ± 0.98^6^	154	0.57 ± 1.31^7^	54	**0.036**^**1**^
**Sum of skinfolds (mm)**	13.4 ± 4.5	124	13.0 ± 3.6	152	15.0 ± 6.2	54	0.170^3,4^
**Waist circumference (cm)**	51.6 ± 7.2	126	51.3 ± 7.4	156	52.8 ± 7.7	55	0.394^1^
**Total energy intake (kcal/day)**	1562 ± 387	121	1469 ± 387	154	1483 ± 456	55	0.145^1^
**Energy intake from lipids (kcal/day)**	462 ± 163^8^	121	410 ± 133^9^	154	416 ± 167^8,9^	55	**0.014**^**1**^
**Energy intake from carbohydrates (kcal/day)**	869 ± 235	121	838 ± 239	154	856 ± 264	55	0.557^1^
**BMI variation 1-4 years (%)**	-9.20 ± 8.96	124	-9.97 ± 8.01	150	-7.46 ± 10.42	53	0.211^5^
**8 years**							
**BMI Z-score**	0.48 ± 1.25^10,11^	114	0.14 ± 1.35^10^	141	0.78 ± 1.60^11^	49	**0.011**^**1**^
**Sum of skinfolds (mm)**	17.7 ± 8.7	114	16.6 ± 7.0	142	22.2 ± 12.8	49	**0.048**^**3,4**^
**Waist circumference (cm)**	57.0 ± 7.0	114	56.1 ± 5.5	142	59.5 ± 8.2	49	0.054^4^
**Total energy intake (kcal/day)**	1557 ± 374	113	1577 ± 347	141	1571 ± 480	49	0.911^1^
**Energy intake from lipids (kcal/day)**	449 ± 155	113	457 ± 139	141	480 ± 202	49	0.495^1^
**Energy intake from carbohydrates (kcal/day)**	890 ± 218	113	911 ± 206	141	871 ± 252	49	0.493^1^
**BMI variation 8-4 years (%)**	6.96 ± 11.21	109	3.60 ± 10.63	136	8.70 ± 13.50	46	**0.015**^**5**^

Abbreviations: *BMI*, body mass index.

^1^ Oneway ANOVA.

^2^ General linear model including the covariate intervention group.

^3^ Ln-transformed variable.

^4^ Kruskal-Wallis test.

^5^ General linear model including the covariates gender, age variation between phase 1 y and phase 4 y and intervention group.

^6,7^ Tukey test T/T x T/A P = 0.537; T/A x A/A P = 0.028; T/T x A/A P = 0.200.

^8,9^ Tukey test T/T x T/A P = 0.013; T/A x A/A P = 0.958; T/T x A/A P = 0.151.

^10,11^ Tukey test T/T x T/A P = 0.118; T/A x A/A P = 0.014; T/T x A/A P = 0.403.

When we analyzed the diet composition, we found a significant difference in the total energy intake from lipids between the three genotypes (P = 0.014). The Tukey post-hoc test revealed that the T/T genotype carriers had a higher mean of lipids intake than the T/A genotype (P = 0.013). When we adjusted the energy intake from lipids for BMI, this association was no longer significant (P = 0.055; data not shown). The total energy intake from carbohydrates was not different between genotypes.

Likewise, at 8 years of age we observed a statistically significant difference in the mean BMI Z-score among the *FTO* genotypes (P = 0.011; Table 2). When the Tukey test was applied, we found that the carriers of the A/A genotype had higher means of BMI than the T/A carriers (P = 0.014). For the other anthropometric parameters at the age of 8, we observed that the sum of skinfolds showed marginally significant differences among the *FTO* genotypes. The percentage of BMI variation between the ages of 4 to 8 also showed significant differences between the genotypes (P = 0.015). The variables related to the dietary profile showed no significant differences between the genotypes at the age of 8.

Longitudinal mixed models including anthropometric and dietary measurements as dependent variables and FTO A/A genotype and sex (this last only when significant) as independent factors are shown in Table 3. In accordance with previous analyses, the A/A genotype was significantly associated with higher BMI Z-score (P = 0.001), waist circumference (P = 0.001) and sum of skinfolds (P = 0.006), but not with total energy intake or energy from lipids or carbohydrates.

**Table 3:** **Coefficients, s.e.s, and *P*-values from the best models for *FTO *rs9939609 predicting anthropometric and dietary variables in the São Leopoldo cohort**

**Dependent variable**	**Effect**	**Coefficient**	**s.e.**	**P-value**
**BMI Z-score**	Intercept	0.531	0.064	<0.001
	FTO (0 = T/?; 1 = A/A)	0.353	0.107	0.001
**Waist circumference**	Intercept	51.6	0.5	<0.001
	FTO (0 = T/?; 1 = A/A)	2.6	0.8	0.001
**Ln Σ skinfolds**	Intercept	2.6	0.1	<0.001
	FTO (0 = T/?; 1 = A/A)	0.1	0.1	0.006
	Sex (0 = F; 1 = M)	-0.1	0.1	0.014
**Total energy intake**	Intercept	893.7	29.7	<0.001
	FTO (0 = T/?; 1 = A/A)	-14.5	36.1	0.689
	Sex (0 = F; 1 = M)	68.3	26.9	0.011
**Energy from lipids**	Intercept	245.9	10.2	<0.001
	FTO (0 = T/?; 1 = A/A)	-1.0	13.4	0.939
	Sex (0 = F; 1 = M)	19.8	10.0	0.048
**Energy from carbohydrates**	Intercept	523.6	14.3	<0.001
	FTO (0 = T/?; 1 = A/A)	-18.2	21.1	0.390

We analyzed the same gene variant in an independent sample of schoolchildren recruited in a nearby city, in the hopes of replicating these findings. The Sapucaia city is located only about 11 km of the first locality and the genotype frequencies of the *FTO* variant here investigated were similar in both samples (χ^2^ = 2.819; df = 2; *P* = 0.244). Significant differences in the mean BMI Z-score and the sum of skinfolds were found among the genotypes (P = 0.029 and P = 0.016, respectively; Table 4). The Tukey test showed that the T/A genotype had higher means for the BMI Z-score (P = 0.025) and the sum of skinfolds (P = 0.020) than did the T/T genotype. The mean waist circumference was not different among genotypes.

**Table 4:** **Mean anthropometric parameters patterns according to *FTO *genotypes in the Sapucaia do Sul sample**

**Phenotypes**	**T/T**		**T/A**		**A/A**		***P***
	**Mean ± SD**	**n**	**Mean ± SD**	**N**	**Mean ± SD**	**n**	
**BMI Z-score**	0.43 ± 1.39^3^	244	0.75 ± 1.33^4^	293	0.70 ± 1.50^3,4^	77	**0.029**^**1**^
**Sum of skinfolds (mm)**	28.8 ± 14.0^5^	245	31.6 ± 13.7^6^	293	32.1 ± 14.3^5,6^	77	**0.016**^**1,2**^
**Waist circumference (cm)**	69.2 ± 11.5	245	70.2 ± 11.8	293	69.1 ± 12.4	77	0.550^1^

Abbreviations: *BMI*, body mass index.

^1^ Oneway ANOVA.

^2^ Statistical tests performed with ln-transformed variable.

^3,4^ Tukey test T/T x T/A P = 0.025; T/A x A/A P = 0.967; T/T x A/A P = 0.298.

^5,6^ Tukey test T/T x T/A P = 0.020; T/A x A/A P = 0.985; T/T x A/A P = 0.127.

The Sapucaia sample was further divided in children (4 to 10 years old, n = 293) and adolescents (11 to 18 years old, n = 322) and the anthropometric measurements were compared among genotypes in these subgroups. There were no significant differences in BMI Z-score, sum of skinfolds or waist circumference in children. In adolescents, the only statistical significant difference was observed in the sum of skinfolds (mean ± SD, T/T = 30.7 ± 14.5 mm; T/A = 34.4 ± 14.8 mm; A/A = 36.2 ± 14.5 mm; *P* = 0.020), although for BMI Z-score, the *P*-value was 0.065. This loss of significance can be due to the smaller sample size when the sample is reduced to about half of its size to perform these analyses.

## Discussion

In the present study, we confirmed the association of the *FTO* variant rs9939609 with body mass index and related phenotypes in Brazilian children and adolescents. To our knowledge, this is the first study regarding this gene and phenotypes related to BMI in Latin American children and adolescents.

The frequency of the minor (A) allele found in Brazilian children was 40%, similar to those reported in European cohorts of children and adolescents [3,21,23] and similar to those reported for Brazilian adults from the same geographic region as ours [24,25].

After the first report identifying the association between the *FTO* gene and obesity-related phenotypes [3], several studies have confirmed the association between rs9939609 and increased BMI in populations of European children and adults [23,26-31] while negative results were found in some studies regarding populations of Oceanic [11], African [32,33] and Asian ancestries [10]. However, other studies have found significant associations in these populations [34-41]. The conflicting results among different ethnic populations are likely the result of varying degrees of linkage disequilibrium between SNPs, which suggests that the underlying causative variant is being tagged differently by rs9939609 in these populations [9,23,32] or that there are different gene-environment interactions. The Brazilian population is, in general, highly admixed with contributions from Europeans, Africans and Native Americans to its gene pool. The Southern Brazilian population, which the present study targeted, differs from this general pattern; its inhabitants are mainly of European descent, with an African contribution estimated at approximately 8 to 13% [42]. The Native American contribution is fairly low. Our results show that the rs9939609 SNP is consistently associated with obesity-related phenotypes in this population, even as early as 4 years old. Although this SNP is located in an intron and several studies have shown a strong genetic disequilibrium in this gene region, it is not possible at this time to rule out rs9939609 as a functional variant [4]. Rather, it is necessary to refine the strength and exact nature of the genetic signal that is functionally related to obesity or related traits [4].

Although several studies have reliably confirmed, to varying degrees, the association between the variation at *FTO*, BMI and related phenotypes [43], there is still some controversy regarding when *FTO* gene function begins to take effect. In our research, the *FTO* gene was not associated with any variable for 1-year-old children. We were only able to find an association of the A/A genotype of rs9939609 and a higher BMI Z-score by the age of 4. This finding agrees with a recent meta-analysis performed on 8 cohorts of European ancestry [43]. In that study, the authors suggested that the variation at *FTO* is associated with a shift in the timing of adiposity rebound; the additive effects of the A allele accelerated developmental age by approximately 2.4% per allele, which corresponded to an early adiposity rebound and a higher BMI later in childhood. According to these data, our longitudinal analysis has shown a greater BMI variation between the ages of 4 and 8 in the A/A subjects.

When we followed the same sample of children until 8 years old, we again found an association between the *FTO* genotypes and the BMI; the A/A homozygotes had higher mean BMI Z-scores than the children carrying one copy of the rs9939609 variant. The A/A genotype was also marginally associated with increased subcutaneous fat, evaluated through the sum of skinfolds. These findings are corroborated by previous childhood [31] and adolescents [44] studies. Furthermore, these results were replicated in an independent sample of schoolchildren between 5 and 18 years of age. In this second sample, the carriers of the T/A genotype possessed a higher BMI Z-score and more skinfolds than the T/T genotype. However, in these older children, the T/A and A/A individuals showed similar means of these two parameters, which diverges somewhat from the results in the first cohort, where the A/A and T/A genotype groups were different. Previous works indicate an additive effect of the A-allele [3,22,43,45]. In all cross-sectional and longitudinal analyses performed, it can be observed that the significant differences regarding BMI are between the T/A and A/A genotypes in the cohort of São Leopoldo, which suggest that, in these young children, there is an recessive effect of the A allele on BMI. However, we cannot exclude the possibility that the difference between the A/A and T/T genotypes may have not been observed due to the moderate sample size evaluated herein or to some environmental or gene-gene interaction that cannot have been addressed with the current approach.

It has been hypothesized that the *FTO* gene has been associated with obesity because it can influence energy homeostasis by having a direct effect on food intake in animal models [46]. A study recently published by Larder et al. (2011) [47] suggests that the increased consumed energy in risk allele carriers is due to a major preference for energy-dense foods, specifically those with a higher fat content, rather than a general increase in the amount of food consumed. However, our data do not seem to corroborate this hypothesis. When we analyzed dietary composition, we observed that the A/A children consumed less energy from lipids by the age of 4. We hypothesized that this reduction in fat consumption in the 4-year-old children with the A/A genotype could be the result of greater parental diet control because this group possessed a higher BMI. In fact, when we adjusted the energy intake from lipids for BMI, the former association was no longer significant. Accordingly, at 8 years old, the association between the dietary composition and the *FTO* genotypes in our sample disappears and, unfortunately, in the independent sample the dietary patterns were not available. As stated previously, the exact role of the *FTO* gene product in the regulation of human energy metabolism is still unclear, and our findings do not suggest a higher total energy intake in the A-allele carriers.

It is known that the *FTO* is expressed in the hypothalamus, mainly in the arcuate nucleus, which is responsible for controlling appetitive behavior [6,48]. The *FTO* variants may also influence macronutrient choice because the arcuate nuclei contains key neuronal populations that make up the central melanocortin pathway, and studies in mice have shown that Fto is expressed within proopiomelanocortin neurons. A reduction in central melanocortin tone has been linked to an increase dietary preference for fat [49]. Thus, it is possible that *FTO* variants are able, either directly or indirectly, to modulate hypothalamic melanocortin activity [47].

A recent study in mice reported the relationship of *FTO* and the epigenetic process. The *FTO* gene has been proposed as a transcriptional coativator that enhances the transactivation potential of the CCAAT/enhancer binding proteins (C/EBPs) from unmethylated as well as methylation-inhibited gene promoters, which suggests a role in the epigenetic regulation of development and maintenance of fat tissue [50]. While the exact function of the *FTO* gene remains to be clarified, it becomes difficult to speculate the mechanism underlying these findings.

There are some limitations to our study. First, the sample size is lower than in other studies; although even under these conditions, we were able to replicate the previous associations of *FTO* SNPs and increased BMI in children and adolescents. Another limitation is that our replication sample data were cross-sectional and detailed food intake data were not available, which precludes deeper analyses in this sample. Furthermore, only 127 (20.7%) of the children of the Sapucaia sample were in the same age range of the São Leopoldo cohort. On the other hand, relevant data are brought by this second sample exactly due to the fact that it comprehends children and adolescents of a higher age and allows us to infer if that the findings in younger children can be extended until the age 18.

## Conclusion

Overall, our work presents new data regarding associations of the *FTO* gene in a previously unexplored population and confirms worldwide data regarding the influence of this gene on the genetic susceptibility for an increased BMI and specific adiposity measures. Furthermore, we were able to show, through a longitudinal analysis that this effect begins around the age of 3 to 4. The exact role of the *FTO* gene in body weight regulation as well as the functionality of the rs9939609 variant remain to be elucidated, but this genetic association is, undoubtedly, the most consistent finding to date.

## Abbreviations

BMI: Body mass index; C/EBPs: CCAAT/enhancer binding proteins; FTO: Fat mass and obesity gene; GWAs: Genome wide association studies; HIV: Human immunodeficiency virus; T2DM: Type 2 diabetes mellitus; SNP: Single nucleotide polymorphism; WHO: World Health Organization

## Competing interests

The authors declare that they have no competing interests.

## Authors’ contributions

CFS genotyped the patients, performed the statistical analyses and wrote the manuscript. MRZ participated of the Sapucaia data and sample collection, DNA extractions and statistical analyses. MRV and PDBC were responsible for all the São Leopoldo cohort selection, following, nutritional evaluation and sample collection. LNR was responsible for all the Sapucaia sample selection, evaluation and sample collection. SA participated in the study design, statistical analyses and manuscript writing. VSM conceived and designed the experiments and coordinated the genotyping, statistical analyses and manuscript writing. All authors read and approved the final manuscript.

## Pre-publication history

The pre-publication history for this paper can be accessed here:

http://www.biomedcentral.com/1471-2350/14/34/prepub
